# Development of CdSe–ZnO Flower-Rod Core-Shell Structure Based Photoelectrochemical Biosensor for Detection of Norovirous RNA

**DOI:** 10.3390/s18092980

**Published:** 2018-09-06

**Authors:** Zhizhong Han, Qinghua Weng, Chaofan Lin, Jinquan Yi, Jie Kang

**Affiliations:** School of Pharmacy, Fujian Medical University, Fuzhou 350122, China; 15870101291@163.com (Q.W.); 15280173200@163.com (C.L.); fjzpyjq@163.com (J.Y.); davidkj660825@163.com (J.K.)

**Keywords:** CdSe–ZnO flower-rod, photoelectrochemical biosensor, norovirous RNA, ion-exchange method

## Abstract

In this work, the CdSe–ZnO flower-rod core-shell structure (CSZFRs) was prepared by ion-exchange method. The surface of CSZFRs was modified by 3-mercaptopropionic acid (MPA), and then the DNA probe was immobilized on the surface via chemical bond between -NH_2_ of DNA probe and -COOH of MPA. Finally, the target norovirous (NV) RNA was combined with the probe according to the principle of complementary base pairing, resulting in a decrease of the photocurrent. The results show that the absorbance spectrum of visible light is enhanced for CSZFRs compared with pure ZnO. Under visible light irradiation, the photocurrent of CSZFRs is up to 0.1 mA, which can improve the sensitivity of the photoelectrochemical (PEC) biosensor. In the measurement range of 0–5.10 nM, the measured concentrations (*c*) have a good linear relationship with the output photocurrent of the biosensor. The linear regression equation is expressed as *I* = 0.03256 − 0.0033*c* (*R*^2^ = 0.99, S/N = 3) with a detection limit of 0.50 nM. Therefore, this work realizes a rapid and sensitive method for the detection of NV RNA.

## 1. Introduction

Norovirus (NV), one genus of the family of Caliciviridae, is one of the main pathogens causing nonbacterial gastroenteritis in humans. It is highly infectious and variable that can lead to serious public health problems especially in people with low resistance [1,2]. There is a high incidence rate, so it is very important to develop rapid and accurate detection techniques for the prevention and control of the transmission of norovirus. Conventional detection methods of NV RNA include immunochromatography [3] and reverse transcription-polymerase chain reaction (RT-PCR) [4,5]. However, they have certain limitations, such as contrasting sensitivities, post-amplification specimen handling, assay complexity, and the requirement for accurate performance tests [6]. Photoelectrochemical (PEC) biosensor is a new biodetection technology with several advantages, such as high sensitivity, good specificity, high analysis speed, low price, and so on. Therefore, PEC biosensors has attracted great attention and widespread application in the field of biology and clinical detection [7,8].

Nano ZnO has been widely used in the field of biosensors due to the advantages of high electron mobility, stable chemical properties, and good biocompatibility [9]. Because of their high electron transport rate and adsorption surface activity, ZnO nanorods and nanoflowers are used to improve the signal transmission rate and enhance the immobilizing effect of the biological molecules in PEC biosensor [10,11]. In addition, the surface of nano ZnO is easy to modify, which provides the conditions to improve ZnO based PEC [12,13,14]. Li et al. prepared ZnO nanorods in flexible polyimide (PI) substrate through hydrothermal method and fixed the glucose oxidase on the surface [15]. The biological enzyme electrode prepared on flexible substrate showed a sensitive current response, which paved a way for the preparation of flexible glucose biosensor. 

However, nano ZnO has lager bandgap, which leads to an only response to the UV region of the solar spectrum and inhibit the utilization of solar energy. The semiconductor quantum dots (QDs) with narrow bandgap can effectively solve this problem [16,17,18,19,20]. QDs, semiconducting nanocrystals with optical effects within a certain range (1–10 nm) have good photoelectric effect, wide and continuous absorption spectra, narrow and symmetrical emission spectra, and good biocompatibility [21,22,23,24]. QDs are widely used in the biology field because of their photoelectrochemical stability and spectral properties [25,26,27,28]. In Nie’s work, QDs were used for cell and tissue sign imaging, which marked the start of QDs application in the biology field [29,30]. 

In this work, CdSe–ZnO flower-rod core-shell nanocable arrays (CSZFRs) were prepared via ion-exchange method and used as a new PEC biosensor for detection of norovirous RNA (NV RNA). The photocurrent is up to mA level. It shows a high sensitivity for the low concentration of NV RNA. In addition, it also has great advantages like convenient use, simple equipment, and fast response. The proposed PEC biosensor exhibits broad prospects and potential applications to be explored in biological detection and analysis respect.

## 2. Experimental

### 2.1. Materials and Reagents

Indium doped tin oxide (ITO) substrate with 15 Ω/sq was obtained from Heptachroma Solar Tech. Zinc acetate dihydrate (Zn(Ac)_2_·2H_2_O), zinc nitrate hexahydrate (Zn(NO_3_)_2_·6H_2_O), ammonium hydroxide (NH_3_·H_2_O) of 25–28%, hexamethy-lenetetramine (HMTA) and NaBH_4_ were purchased from Sino Chem. Reagent. Poly-ethylenimine (PEI) and N-hydroxysulfosuccinimide sodium salt (NHS) were obtained from Aladdin Reagent. Se powder was obtained from Shanghai Meixing Chemical Co., Ltd. (Shanghai, China) Cd(NO_3_)_2_ and 3-mercaptopropionic acid (MPA) purchased from Alfa Aesar. N-(3-Dimethylaminopropyl)-N’-ethyl carbodiimide hydrochloride (EDAC) was obtained from Life Science Products & Services. Target complimentary NV RNA of genogroup II (G11) and DNA probe were purchased from Sangon Biotech (Shanghai, China).

DNA sequence: 5′-/NH_2_-/GCGACGAATTAGCTTGTATGATGTCGTCGC-3′

RNA sequence: 5′-GACAUCAUACAAGCUAAUUC-3′

### 2.2. Preparation of CdSe Sensitized ZnO Flower-Rods

CSZFRs were prepared by ion exchange method according to our previously reported method [31]. ZnO flower-rods arrays (ZnO FRs) were prepared via solution-based method. Firstly, ITO substrates were washed with different cleaning reagent and dried in an oven. Secondly, the substrates were seeded by spin coating (1000 rpm, 39 s) with 5 mM Zn(Ac)_2_·2H_2_O in ethanol, followed by thermal decomposition at 300 °C for 20 min. Thirdly, the seeded substrates were placed in an aqueous solution containing 25 mM Zn(NO_3_)_2_·6H_2_O, 12.5 mM HMTA, 5 mM PEI, and 0.35 M NH_3_·H_2_O at 85 °C for 4 h. Finally, the as-prepared ZnO samples were dried in oven and then calcined at 450 °C for 30 min.

ZnO FRs were immerged in a Se^2−^ ion solution (6.0 mM) prepared by reacting Se powder with NaBH_4_ and kept at 50 °C for 20 min under the condition of nitrogen gas. This process was repeated for two times to get a desirable thickness of ZnSe. The arrays of ZnO/ZnSe nanocables then immersed into Cd(NO_3_)_2_ aqueous solution (0.1 M) at 90 °C for 8 h, then ZnSe was converted to CdSe and CSZFRs were formed. At last, CSZFRs were dipped in MPA solution at 90 °C for 2 h.

### 2.3. Construction of CdSe–ZnO Flower-Rods Based PEC Biosensor

EDAC and NHS solutions were used to activate the carboxylate groups on the surface of CSZFRs at 37 °C for 30 min. Then DNA probe (100 nM, 6 μL) was added onto CdSe–ZnO/ITO and activated at 37 °C for 1 h (marked as CSZFR@DNA). The actual molar amount of DNA probes on the electrode surfaces is about 0.585 pmol, which was measured with the method of spectrophotometry. Different concentrations of 15 μL NV RNA, which were prepared using pH = 8 Tris-EDTA buffer, were hybridized with the DNA oligonucleotide sequence in the dark for 5 min (marked as CSZFR@RNA). 

### 2.4. Material Characterizations

Crystal structures of CSZFRs were characterized by powder X-ray diffraction (XRD, X’pert Pro MPD X, Almelo, The Netherlands) and field-emission scanning electron microscopy (FE-SEM, Hitachi S4800, Tokyo, Japan). The diffuse reflectance spectra were measured on a Shimadzu UV-2500 UV-Visible spectrophotometer (Shimadzu, Tyoto, Japan). Fluorescence measurement was performed at room temperature on a Cary Eclipse fluorescence spectrometer (Agilent, Santa Clara, CA, USA) in a 300 μL quartz cuvette.

### 2.5. Photoelectrochemical Measurements

The photocurrent measurement of the PEC biosensor was carried out using an electrochemical workstation (CHI 660D, CH Instrument Company, Shanghai, China). All electrochemical analyzes were performed using a conventional three-electrode system under the irradiation of xenon lamps (HSX-UV300, NBeT, Beijing, China), where UV light was cut with UV cut 400 filter. The saturated Ag/AgCl electrode was used as the reference electrode, the platinum wire electrode as the counter electrode and the as-prepared CdSe–ZnO/ITO or ZnO/ITO as the working electrode. The electrolyte solution was 0.1 M phosphate buffer solution (PBS). The impedance spectrum of the PEC biosensor was measured using the same electrochemical workstation.

For the experiment of recovery, the human serum was diluted 1000 times, and then added different molars of NV RNA into the diluted serum. The operation was the same as above PEC measurement and repeated 5 times for each sample (*n* = 5).

## 3. Results and Discussion

**Figure 1:**
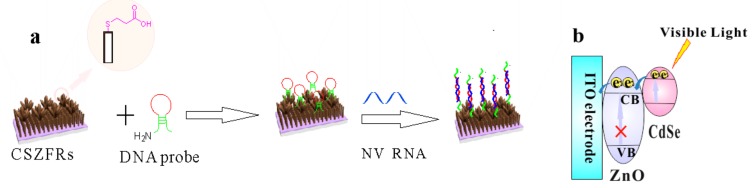
Schematic illustration of (**a**) the synthesis process and (**b**) electron transfer of CSZFRs based PEC biosensor.

As Figure 1a shown, DNA probes are combined with CSZFRs by chemical bonds between -NH_2_ of the DNA probe and -COOH of the MPA, then NV RNA was hybridized with DNA via complementary base paring, leading to a rapid reduction of the photocurrent response. DNA probe and NV RNA are insulators, which hinder the charge transfer due to their high resistance. Because of the steric hindrance effect, DNA and NV RNA can restrain the transmission of electrons and induce a reduction in PEC signals. The narrow bandgap CdSe QDs can overcome the defect of lager bandgap ZnO to absorb the visible region of solar spectrum and enhance the utilization of solar energy. Figure 1b shows that the valence band (VB) and conduction band (CB) of CdSe are both higher than those of ZnO. Therefore, under visible light irradiation, the photogenerated electrons can transfer from VB of CdSe to CB, and then transfer to CB of ZnO, finally transfer to the surface of ITO glass to generate photocurrent.

**Figure 2:**
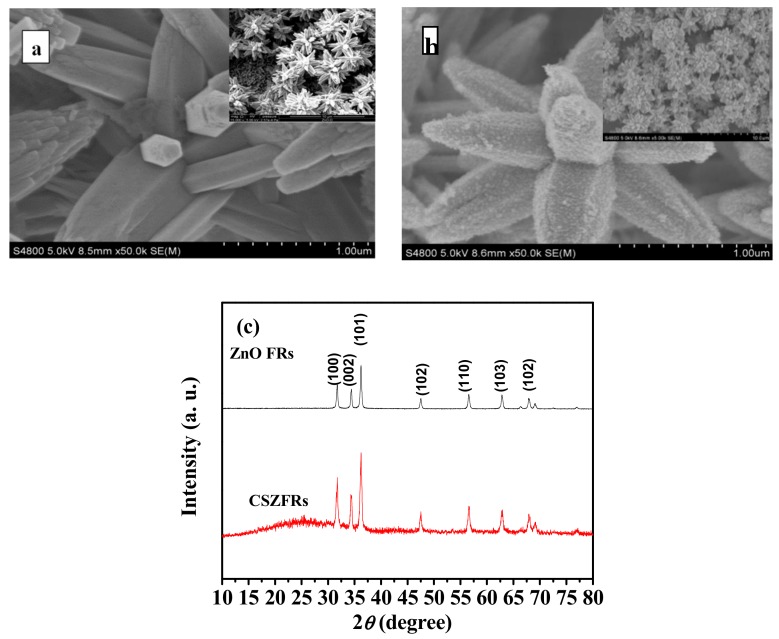
FESEM images of (**a**) ZnO FRs and (**b**) CSZFRs. The insets of (**a**) and (**b**) are images with lower magnification; (**c**) XRD patterns of CSZFRs and ZnO FRs.

Figure 2a,b show FE-SEM images of the as-prepared ZnO FRs and CSZFRs on ITO substrate. The pure ZnO has a flower and rod like structure with smooth surface on a rod array (Figure 2a and its insert). While CSZFRs have a rougher surface and there are many small particles with uniform distribution without obvious aggregation, which indicates that CdSe QDs cover the ZnO substrate very efficiently (Figure 2b). Further, the pattern of CSZFRs is consistent with ZnO FRs, which shows that the core-shell structure prepared by ion-exchange method is not destroyed (inset of Figure 2b).

Figure 2c displays the XRD patterns of the as-prepared samples. The diffraction peak intensity of CSZFRs is lower, indicating that ZnO is covered with CdSe nanoparticles successfully. It shows that the grain is well developed. Three distinct peaks at 31.8°, 34.5°, and 36.3° are observed in both patterns, which are representing (100), (002), and (101) crystal planes of ZnO (JCPDS36-1451), respectively. The three strongest diffraction peaks of CdSe are located at 2*θ* = 25.5°, 42.0°, and 49.5° (JCPDS19-0191). The XRD pattern of CSZFRs has a broad peak between 20–30° and the diffraction peaks of 42° and 49.5° are not observed, which is due to the smaller size and lower amount of CdSe QDs. The result matches well with FESEM image of CdSe–ZnO.

**Figure 3:**
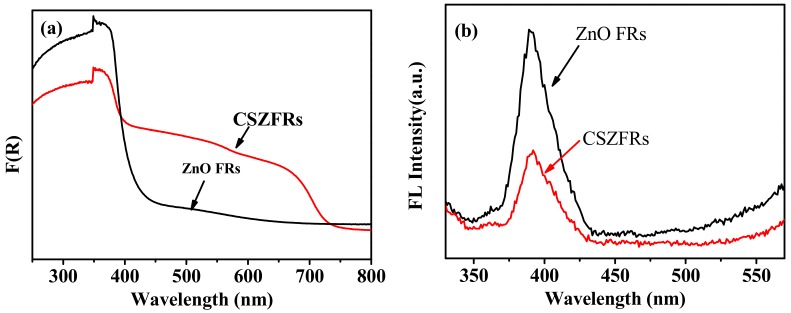
(**a**) UV-Visible diffuse reflectance spectra and (**b**) Fluorescence spectra of CSZFRs and ZnO FRs.

Figure 3a exhibits UV-Visible diffuse reflectance spectra. It is found that the peak intensity of pure ZnO between 250 nm and 400 nm is greater than that of CSZFRs, which indicates that CdSe is bonded to ZnO surface and the CSZFRs core-shell structure is successfully constructed. However, the peak intensity of CSZFRs is greater than that of pure ZnO between 400 and 700 nm. In addition, the absorption band red shifts to about 730 nm. It indicates that CdSe QDs greatly enhances the absorption of ZnO in the visible region.

It can be seen from Figure 3b that the fluorescence intensity of CSZFRs is smaller than that of ZnO FRs and an obvious quenching phenomenon occurs. The stronger fluorescence intensity of ZnO FRs implies that its electron-hole recombination is strong, which will produce a large number of fluorescence. The quenching effect of CSZFRs demonstrates that CdSe–ZnO composites facilitate the separation of a photogenerated electron-hole, which is beneficial to the enhancement of photocurrent. The cover of CdSe may improve the surface defect of ZnO, suppressing the recombination of electron and surface vacancy. Moreover, the band alignment of CdSe and ZnO also enable the separation of photogenerated electron and hole. Figure 3 elucidates that CSZFRs can expand the absorption of visible light and inhibit the recombination of photogenerated electron-hole pair, so that the prepared biosensor can enhance photocurrent and sensitivity under visible light irradiation.

**Figure 4:**
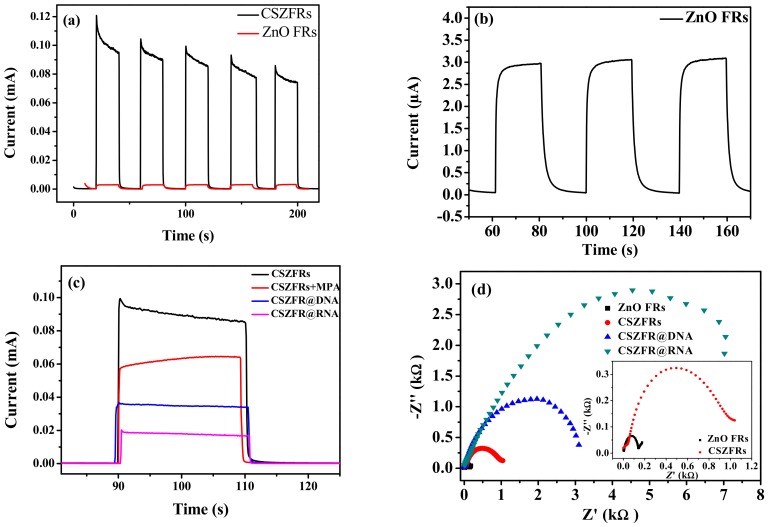
(**a**) Photocurrent of CSZFRs and ZnO FRs; (**b**) Photocurrent of pure ZnO FRs; (**c**) The photocurrents of different samples; (**d**) Electrochemical impedance spectroscopy for different samples. The inset of (**d**) is EIS of ZnO FRs and CSZFRs.

According to Figure 4a, under visible light, the photocurrent of CSZFRs is greatly improved and reached up to about 0.1 mA, but the photocurrent of pure ZnO is only about 3.0 μA (Figure 4b). The result indicates that CdSe QDs have a certain sensitizing effect, which greatly improves the photocurrent of ZnO. However, when 3-mercaptopropionic acid were combined onto CSZFRs, the photocurrent is drastically declined (Figure 4c). In comparison with CSZFRs, the photocurrent responses of the samples what are added with DNA and RNA are reduced by 10 times. For the decrease of photocurrent one reason is the steric hindrance and insulation of DNA and RNA. Another may be that the charge transfer rate is inhibited after the hybrid of DNA probe and NV RNA [32].

Electrochemical impedance spectroscopy is an effective method to explain the information of impedance change and the characteristics of the electrodes in the modification process, in which the semicircle diameter represents the impedance of electron transfer at the interface of the dominant electrode *R*_ct_. As Figure 4d shown, the impedance value increase from about one hundred ohm to seven thousand ohm. The insert of Figure 4d shows that the bare ZnO FRs electrode presents a small semicircle, which is a rapid transfer of [Fe(CN)_6_^3−/4−^] electrons. After loading the quantum dots, the impedance of CSZFRs increases. Additionally, the impedance value of CSZFRs after adding the probe DNA and the target RNA is much larger than that of the bare ZnO FRs and CSZFRs, which is mainly due to the DNA and RNA insulators. Moreover, the addition of DNA and RNA also makes its steric hindrance larger. And the charge transfer rate is inhibited after the hybrid of DNA probe and NV RNA. This result is consistent with Figure 4c.

**Figure 5:**
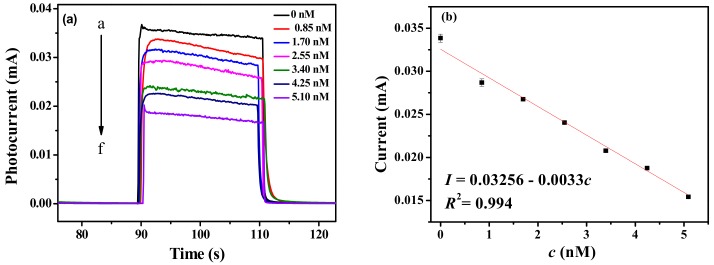
(**a**) Photocurrents of the as-prepared PEC biosensor based on CSZFRs with different concentrations of NV RNA; (**b**) Linear relationship graph.

As shown in Figure 5a, the photocurrent has variety with different concentrations of NV RNA under visible light irradiation. As the concentration of NV RNA increases, the photocurrent decreases. It is attributed to the insulation of RNA and its larger steric hindrance. Linear relationship between the photocurrent and the concentration of NV RNA from 0–5.10 nM is shown in Figure 5b. The linear equation is *I* = 0.03256 − 0.0033*c* (*R*² = 0.99, S/N = 3) and the detection limit is 0.50 nM. The value of limit of detection (LOD) is given by the equation LOD = −3 σ/s, where σ is the standard deviation of the blank measures, and s is the slope of linear equation. The PEC biosensor based on pure ZnO was also used for detecting RNA under visible light (Figure S1). However, the photocurrent is only in the μA level and the difference of photocurrent is only 0.55 μA between the detection of 0.85 and 5.10 nM. Therefore, the PEC biosensor based on CdSe–ZnO has higher selectivity for the detection of NV RNA.

In order to verify the applicability of a PEC biosensor for NV RNA detection, we performed a standard recovery experiment in normal human serum. The results are shown in Table 1. The recoveries for the high, medium, and low concentrations are from 100.88% to 103.22% with the average recovery rate of 101.97%. The relative standard deviation (RSD) values for high, medium, and low concentrations are 8.58%, 5.88%, and 2.29%, respectively. Repeat five times for each sample (*n* = 5). The results show that the developed PEC biosensor has good applicability.

## 4. Conclusions

As the results shown, CSZFRs were successfully prepared by the ion-exchange method. UV-Visible diffuse reflectance spectra and fluorescent spectra show that CdSe can effectively enhance the visible light absorption of ZnO, promote the separation of a photogenerated electron-hole and improve the efficiency of photoelectric conversion. The CSZFRs based PEC biosensor has good detection performance for NV RNA. The photocurrent has a good linear relationship with the concentration of NV RNA in the range of 0–5.10 nM with a detection limit of 0.50 nM. The results reflect that the constructed PEC biosensor based on CSZFRs which is sensitive and fast for detecting NV RNA can be used for the detection of other biomolecules.

## Figures and Tables

**Table 1:** Spike recovery test for the detection of NV RNA in normal human serum.

Samples	Addition (nM)	Found (nM, *n* = 5)	Recoveries (%)	Average Recovery (%)	RSD (%)
**1**	**1.70**	**1.73**	**101.80**	**101.97**	**8.58**
**2**	**3.40**	**3.43**	**100.88**		**5.88**
**3**	**5.10**	**5.25**	**103.22**		**2.29**

## Supplementary Materials

The following are available online at http://www.mdpi.com/1424-8220/18/9/2980/s1, Figure S1: Photocurrents of PEC biosensor based on ZnO with different concentrations of NV RNA.
