# Supplementary material for: Development of CdSe–ZnO Flower-Rod Core-Shell Structure Based Photoelectrochemical Biosensor for Detection of Norovirous RNA
Source: Sensors (Basel). 2018 Sep 6;18(9):2980. doi: 10.3390/s18092980 (PMC6164258; doi:10.3390/s18092980)
Supplement: Supplementary file 1 [file sensors-18-02980-s001.pdf]

sensors

MDPI

Development of CdSe–ZnO Flower-Rod Core-Shell Structure based Photoelectrochemical Biosensor for Detection of Norovirous RNA

Zhizhong Han*, Qinghua Weng, Chaofan Lin, Jinquan Yi and Jie Kang
School of Pharmacy, Fujian Medical University, Fuzhou, Fujian 350122, P R China; 15870101291@163.com(Q.W.); 15280173200@163.com(C.L.); fjzpyjq@163.com(J.Y.); davidkj660825@163.com(J.K.)
* Correspondence: zzhan@fjmu.edu.cn

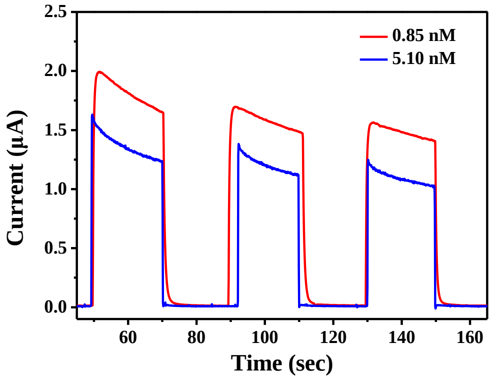

Figure S1. Photocurrents of PEC biosensor based on ZnO with different concentrations of NV RNA.
